# Influence of adult attachment insecurities on parenting self-esteem: the mediating role of dyadic adjustment

**DOI:** 10.3389/fpsyg.2015.01461

**Published:** 2015-09-24

**Authors:** Vincenzo Calvo, Francesca Bianco

**Affiliations:** Department of Philosophy, Sociology, Pedagogy and Applied Psychology, University of PadovaPadova, Italy

**Keywords:** adult attachment, dyadic adjustment, parenting self-esteem, parenting self-efficacy, parenting satisfaction

## Abstract

**Background:** Parenting self-esteem includes two global components, parents’ self-efficacy and satisfaction with their parental role, and has a crucial role in parent–child interactions. The purpose of this study was to develop an integrative model linking adult attachment insecurities, dyadic adjustment, and parenting self-esteem.

**Methods:** The study involved 118 pairs (236 subjects) of heterosexual parents of a firstborn child aged 0–6 years. They were administered the Experiences in Close Relationships-Revised (ECR-R) questionnaire, the Dyadic Adjustment Scale, and the Parenting Sense of Competence Scale.

**Results:** Path analysis was used to design and test a theoretical integrative model, achieving a good fit with the data. Findings showed that dyadic adjustment mediates the negative influence on parenting self-efficacy of both attachment anxiety and attachment avoidance. Parenting satisfaction is positively influenced by parenting self-efficacy and negatively affected by child’s age. Attachment anxiety negatively influences parenting satisfaction.

**Conclusion:** Our findings are in line with the theoretical expectations and have promising implications for future research and intervention programs designed to improve parenting self-esteem.

## Introduction

It has been recognized in both developmental and clinical research that parents’ cognitions and beliefs about parenting have a crucial role in parent–child interactions (Dix and Grusec, 1985; Johnston, 1996; Bugental and Johnston, 2000; Rubin and Chung, 2006), and relate to virtually every aspect of children’s developmental accomplishments (Sigel and McGillicuddy-Delisi, 2002). Parents’ cognitions and beliefs (i.e., their knowledge, values, attitudes, and goals) can have numerous functions (Bornstein, 2002), among which they may generate and shape parental behavior, and help to organize parenting activities (Murphey, 1992; Darling and Steinberg, 1993; Teti and Candelaria, 2002).

One form of parental cognition that has received increasing attention is parenting self-esteem, also known as parenting sense of competence. By definition, this concept encompasses two global, closely related components (Johnston and Mash, 1989): parents’ perceived self-efficacy in their parental role, and the satisfaction they derive from parenting (Sabatelli and Waldron, 1995; Coleman and Karraker, 1997; Bugental et al., 1998; Ohan et al., 2000; Jones and Prinz, 2005; Carpenter and Donohue, 2006; Nunes et al., 2014).

Self-efficacy is about people’s belief in their ability to achieve their goals (Bandura, 1997), and in the context of parenting this means how confident people feel about their capacity to deal competently with difficult childrearing situations. It stems from parents’ cognitions and self-perceptions about how skillfully they accomplish tasks related to parenting (de Montigny and Lacharité, 2005; Jones and Prinz, 2005; Farkas and Valdés, 2010), and positively influence their children, fostering the latter’s adjustment and development (Ardelt and Eccles, 2001). In other words, parenting self-efficacy refers to an instrumental dimension of parenting, notably the degree to which parents feel competent, capable of solving problems, and familiar with the demands of parenting (Johnston and Mash, 1989). Parenting satisfaction indicates a more affective dimension, reflecting the degree to which parents feel frustrated, anxious, gratified and motivated in their parenting role (Johnston and Mash, 1989).

Efficacious parenting beliefs are often associated with greater competence in performing parenting tasks: parents who feel more competent exhibit a greater confidence in acquiring and exercising effective parenting skills, strategies, and types of behavior than parents who feel less competent (Jones and Prinz, 2005). Feeling competent as a parent influences the quality of maternal adaptation during the transition to parenthood (Ngai et al., 2007), the care that parents give to their newborn (Teti and Gelfand, 1991; Tucker et al., 1998; de Haan et al., 2009; Dumka et al., 2010). When parents feel competent, they are likely to use more effective parenting practices, developing more secure, warm, and involving interactions with their child (Coleman and Karraker, 1997; Shumow and Lomax, 2002; Jones and Prinz, 2005). Parents who feel effective have also proved better able to provide an adaptive, motivating and nurturing childrearing environment (Locke and Prinz, 2002). To be more specific, mothers who feel efficacious and competent in their role as parents are more responsive (Parks and Smeriglio, 1986; Schellenbach et al., 1992) and less punitive, and their developmental expectations are more appropriate (East and Felice, 1996). They are also more strongly motivated to engage in further interactions that, in turn, provide them with additional opportunities to interact positively with their infants (Teti et al., 1996). Research has demonstrated that parenting self-esteem is a protective factor in the mother-child relationship, mediating the negative effects of maternal depression and temperamental offspring (Teti and Gelfand, 1991; MacPhee et al., 1996; Gondoli and Silverberg, 1997). On the other hand, a scarce confidence in one’s parenting skills is associated with frustration and irritation, and a less supportive behavior (Bugental et al., 1984; Sanders and Woolley, 2005; de Haan et al., 2009), which increases the risk of the offspring developing externalizing problems (Hill and Bush, 2001; Sanders and Woolley, 2005; Dishion and Patterson, 2006; Grusec and Hastings, 2007; Prinzie et al., 2010), and delinquent behavior in adolescent age (Bogenschneider et al., 1997).

In the light of the apparent importance of parenting self-esteem in parent–child relationships and children’s well-being (Teti and Gelfand, 1991; Tucker et al., 1998; de Haan et al., 2009; Dumka et al., 2010), it is crucial to investigate and clarify which factors influence the development of its cognitive and emotional components, such as parenting self-efficacy and parenting satisfaction (Sevigny and Loutzenhiser, 2010).

A growing body of empirical research shows that adult attachment profoundly influences parents’ behavior, emotions, and cognitions (Jones et al., 2015). From the standpoint of attachment theory, parenting behavior, cognitions, and emotions are conceptualized as serving the caregiving bio-behavioral system, and thought to be influenced and shaped by previous experiences with caregivers in earlier phases of development (Cassidy and Shaver, 1999; Mikulincer and Shaver, 2007). Caregiving is seen as a primary component of parenting behavior, but also as a key constituent of romantic and marital relationships, and of all forms of prosocial behavior (Mikulincer and Shaver, 2007). Secure working models of attachment are thought to promote and sustain effective caregiving and parenting self-esteem (Mikulincer and Shaver, 2007).

In the tradition of attachment theory, numerous studies have investigated the connections between parenting characteristics and adult attachment, using both interview-based attachment measures, such as the Adult Attachment Interview (AAI; George et al., 1984, 1985, 1996), and also – more recently and with growing interest – self-report questionnaires designed to assess attachment styles, dimensions, and orientations (Jones et al., 2015).

Only relatively few studies have addressed the influence of adult attachment styles on such components of parenting self-esteem as parenting self-efficacy and parenting satisfaction, yielding a complex and variable picture. Some early studies provided preliminary support for the association between adult attachment orientations and parenting self-efficacy (Coleman and Karraker, 1997). Caldwell et al. (2011) studied the relationships between forms of maltreatment, adult attachment dimensions (anxiety and avoidance), maternal depression, and parenting self-efficacy in a group of at-risk mothers: they found that attachment anxiety has an indirect effect on parenting self-efficacy, mediated by maternal depressive symptoms, whilst the direct link between attachment anxiety and parenting self-efficacy was not significant. Similarly, Kohlhoff and Barnett (2013) examined the role of adult attachment and depression as predictors of parenting self-efficacy in a sample of primiparous mothers during the first year after childbirth: their results showed that both attachment anxiety and attachment avoidance have a significant indirect effect on parenting self-efficacy and this link is mediated by the presence of maternal major depression. Only attachment anxiety had a significant but moderate, direct relationship with parenting self-efficacy, after taking the mediating effect of depression into account. Another study explored the connections between fathers’ romantic attachment style, as coded by means of the Hazan and Shaver (1987) three-category measure of attachment, parenting beliefs and the offspring’s attachment security (Howard, 2010): consistently with expectations, fathers who rated themselves as secure scored higher for parenting efficacy and had a better knowledge of their child’s development.

As for the other dimension of parenting self-esteem – parenting satisfaction – a recent review (Jones et al., 2015) examined the literature concerning the links between attachment styles and parenting satisfaction, generating a rather inconsistent picture. As expected, La Valley and Guerrero (2012) found that security was associated with more parenting satisfaction. Along the same lines, attachment avoidance correlated with less parenting satisfaction in four studies (Cohen and Finzi-Dottan, 2005; Rholes, 2006; Cohen et al., 2011; Vieira et al., 2012). One of these four studies only found this link for mothers (Cohen and Finzi-Dottan, 2005), however, and Vieira et al. (2012) only identified an indirect effect of attachment on parenting satisfaction, mediated by work-family conflict. Findings concerning attachment anxiety are less convincing: only Cohen et al. (2011) reported a negative relationship between anxiety and satisfaction (as expected); Rholes (2006) found no significant relationship between the two; Vieira et al. (2012) found both a positive direct effect of attachment anxiety on satisfaction and an indirect path linking anxiety with less parenting satisfaction via more severe work-family conflict; and (contrary to expectations) Lau and Peterson (2011) found no significant associations between attachment style and parenting satisfaction.

Taken together, these results provide some support for links between adult attachment styles and parenting self-efficacy, but these links are likely to be indirect and mediated by other relevant variables; and the picture concerning parenting satisfaction is unclear. More research is needed on these topics to further investigate the association between attachment styles and parenting self-esteem in the general normative (non-clinical) population. The issue is important, given that sociological theory on family stress and role strain point to stress levels as a moderating variable that significantly affects the links between marriage quality, parent–child relationship, and parenting (Erel and Burman, 1995). Indeed, six out of the eight above-mentioned studies examined parents dealing with stressful life circumstances, such as mothers on residential programs for early parenting difficulties (Kohlhoff and Barnett, 2013), at-risk mothers (Caldwell et al., 2011), war veteran fathers (some of them suffering from acute combat-induced stress reaction and post-traumatic stress disorder; Cohen et al., 2011), couples in the first year after divorce (Cohen and Finzi-Dottan, 2005), parents of children with Asperger syndrome (Lau and Peterson, 2011), or parents in the early months of their transition to parenthood (Rholes, 2006).

The extant literature has yet to investigate the mediating role of couple quality and dyadic adjustment in influencing parenting self-esteem, in families where both parents are present. A considerable amount of theoretical and experimental data suggests that the quality of the couple’s relationship might be a salient mediator between adult attachment and parenting self-esteem. Empirical research has shown that adult attachment has a direct impact on couple quality and dyadic adjustment (Collins and Read, 1990; Carnelley et al., 1994, 1996; Collins, 1996; Frazier et al., 1996; Jones and Cunningham, 1996; Whisman and Allan, 1996; Cozzarelli et al., 2000; Frei and Shaver, 2002; Schmitt, 2002; Steiner-Pappalardo and Gurung, 2002; Shi, 2003; Kachadourian et al., 2004; Sumer and Cozzarelli, 2004; Williams and Riskind, 2004; Shaver et al., 2005), and that parental beliefs are strongly influenced by the quality of the relationship with the other parent (Belsky, 1984; Goldberg and Easterbrooks, 1984; Cox et al., 1989; Howes and Markman, 1989; Simons et al., 1992, 1993; Kerig et al., 1993; Erel and Burman, 1995; Cowan et al., 1996; Holloway et al., 2005; Schoppe-Sullivan et al., 2007; Suzuki, 2010). Moreover, Millings et al. (2012) found that attachment orientations have a significant indirect effect on parenting styles, mediated by responsive caregiving to partner.

In short, the literature highlights significant theoretical, conceptual and empirical links between adult attachment orientations, couple quality and parenting self-esteem. In particular, it is apparent that dyadic adjustment can mediate the influence of adult attachment on parenting self-efficacy. At the same time, there is contrasting evidence on the possible direct effect of attachment anxiety on parenting satisfaction, and this issue warrants further investigation.

The aim of the present cross-sectional research was therefore to extend our understanding of the effects of attachment on parenting self-esteem by developing an integrative model linking attachment insecurities (i.e., attachment anxiety and attachment avoidance) to parenting self-efficacy and parenting satisfaction, taking into account the possible role of dyadic adjustment with the partner as a mediator variable. Drawing from attachment theory and past empirical work, we consequently hypothesized a path analytical model of influences, postulating: (a) a direct effect of attachment orientations (attachment anxiety and attachment avoidance) on parenting self-efficacy and/or parenting satisfaction; and (b) a mediated influence of attachment orientations on parenting self-efficacy and parenting satisfaction, via dyadic adjustment. In particular, our hypotheses were that: (a) higher levels of attachment insecurity are directly associated with lower levels of parenting self-esteem; and (b) attachment insecurity negatively influences dyadic adjustment, which in turn reflects on parenting self-esteem. Finally, in accordance with Johnston and Mash (1989), we included in our model a path for the influence that links parenting self-efficacy with parenting satisfaction, hypothesizing that lower levels of parenting self-efficacy may result in less parenting satisfaction. The literature on parental cognitions has shown that beliefs concerning self-efficacy (specifically in the parenting domain) are a powerful variable explaining a significant portion of the variance observed in parenting skills and satisfaction (Coleman and Karraker, 1997). According to Bandura (1982), beliefs concerning their self-efficacy influence people’s way of thinking and determine individuals’ motivations and behavior. Parents with a strong sense of self-efficacy can be more at ease and effective in dealing with the everyday difficulties of being a parent, and this positively influences their satisfaction with their role. Vice versa, a weak sense of self-efficacy may negatively influence parenting practices, causing anxiety, depression, and stress, and reducing parenting satisfaction (Coleman and Karraker, 1997).

**FIGURE 1:**
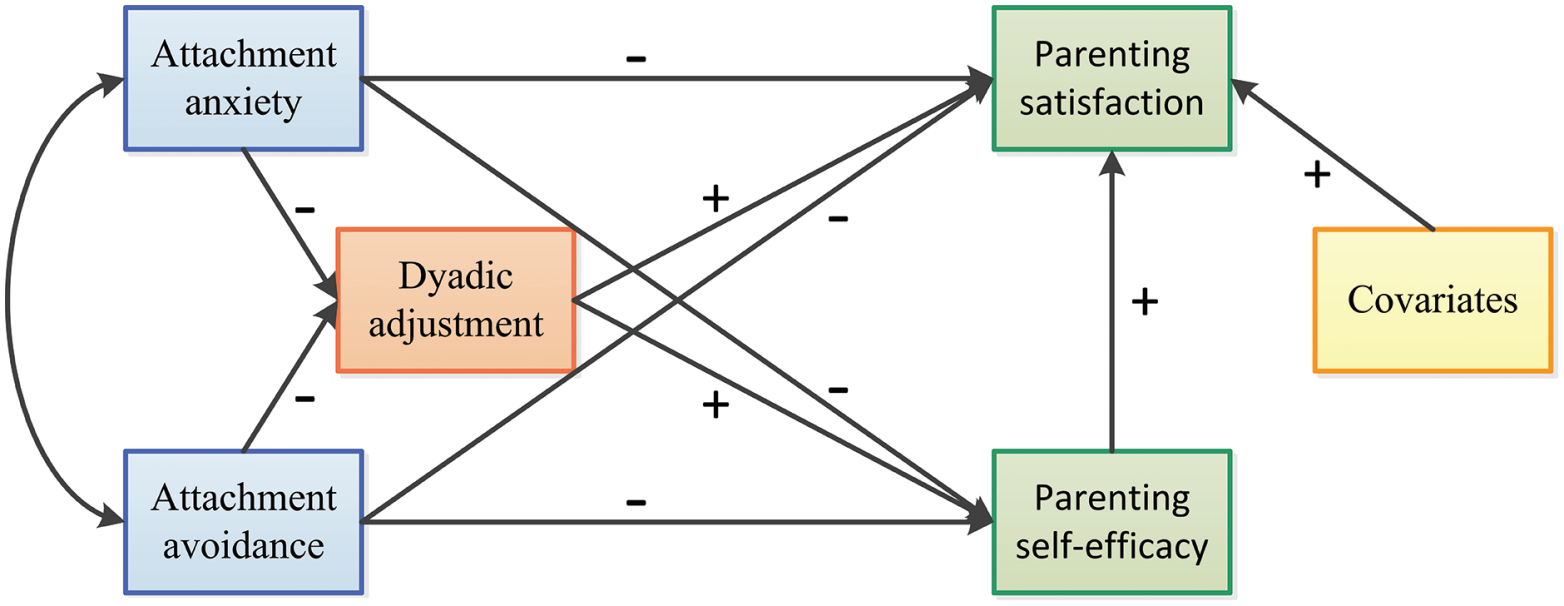
**Theoretical model linking adult attachment, dyadic adjustment, parenting self-esteem.** Plus and minus signs indicate the hypothesized direction of the proposed paths.

Our theoretical integrative model is outlined in **Figure 1**.

Some studies found significant gender-related differences in parenting self-esteem, suggesting that fathers tend to have higher levels of parenting satisfaction than mothers (Johnston and Mash, 1989; Rogers and Matthews, 2004; Gilmore and Cuskelly, 2009). Since mothers and fathers experience at least some parenting processes differently (Bretherton et al., 2005; Gamble et al., 2007; Gilmore and Cuskelly, 2009), we used a multiple group analysis without any specific *a priori* hypothesis to test the extent to which the proposed theoretical model is consistent across genders.

## Materials and Methods

### Participants and Procedure

The study group included 118 pairs (236 subjects) of heterosexual parents with a firstborn child aged 0–6 years. In most cases, the couples had only one child (87 couples, 73.7%), while 30 couples had two (25.4%), and one couple had three (0.8%). The firstborn children were a mean 2.58 years of age (*SD* = 22.27 months; range: 1–72 months); 52 (44.1%) of them were females, and 66 (55.9%) males.

All parents were Caucasian. The mean age of the mothers was 33.51 years (*SD* = 5.54; range: 19.10–47.16 years) and for the fathers it was 36.19 years (*SD* = 5.67; range: 24.54–51.15). The mean duration of the couples’ relationships was 9.20 years (*SD* = 4.29; range: 2–22), and they had been living under the same roof for a mean 5.25 years (*SD* = 3.15; range: 0.5–22). The parents’ formal education had lasted a mean 14.90 years (*SD* = 3.08) for the mothers, and 13.94 years (*SD* = 3.41) for the fathers.

The couples included in the study were enrolled using a chain sampling method. They were invited to participate in a study on the relationships between adult attachment, couple adjustment, and parenting self-esteem. The inclusion criteria were: (1) having a firstborn child aged 0 to 6; (2) being married to or living with the child’s other parent. A psychologist administered all the questionnaires to both parents after informing participants about the aims of the study and asking them to give their written informed consent. The whole procedure took approximately 30 min. The study was approved by the Ethical Committee for the Psychological Research of the University of Padova.

### Instruments and Measures

All participants independently completed the following self-report questionnaires to measure adult attachment, dyadic adjustment, and parenting self-esteem.

#### Adult Attachment

The Experiences in Close Relationships-Revised (ECR-R; Fraley et al., 2000b) questionnaire is a self-report measure of adult attachment. It consists of 36 items, scored on a seven-point Likert scale ranging from 1 (strongly disagree) to 7 (strongly agree). The ECR-R assesses two dimensions of attachment (18 items for each scale): (a) attachment anxiety, which reflects variability in fear of abandonment and sensitivity to issues relating to rejection and loss (Fraley et al., 2000a); and (b) attachment avoidance, which reflects the degree of the individual’s discomfort with intimacy, closeness, and dependence. Higher mean scores indicate greater degrees of anxiety and/or avoidance, and consequently lower levels of attachment security, which is therefore conceptualized as a low degree of attachment anxiety and/or avoidance. Individuals with low scores for both dimensions are willing and able to use their attachment figures as a safe haven during times of distress and danger, and as a secure base from which to explore their worlds. The scores for the two ECR-R dimensions were not used to assign participants to specific attachment categories (i.e., secure, fearful, dismissing, preoccupied) because it has been suggested that individual variation in attachment is modeled better using dimensions rather than categories (Fraley and Waller, 1998; Fraley and Spieker, 2003a,b; Roisman et al., 2007). The Italian version of the ECR-R has demonstrated good psychometric properties in terms of internal consistency, factorial and concurrent validity (Calvo, 2008; Busonera et al., 2014). In the present study, Cronbach’s alpha reliability value was 0.82 for the attachment anxiety score, and 0.86 for the attachment avoidance score.

#### Dyadic Adjustment

The Dyadic Adjustment Scale (DAS; Spanier, 1976) is a 32-item self-report questionnaire assessing marital dyadic adjustment, under four headings: dyadic consensus (13 items), dyadic satisfaction (10 items), dyadic cohesion (five items), and affective expression (four items). Dyadic consensus is the degree to which the couple agrees on matters of importance to the relationship, such as handling family finances or making major decisions. Dyadic cohesion is the degree of closeness and shared activities experienced by the couple. Dyadic satisfaction refers to the degree to which the partners are satisfied with their relationship. Affective expression concerns the demonstrations of affection and sexual relationships. For the purposes of the present study, we only used the DAS total score, computed as the sum of the four subscales, as a measure of overall dyadic adjustment. The Italian version of the DAS has demonstrated an adequate internal consistency and factorial structure (Gentili et al., 2002). In this study, Cronbach’s alpha coefficients for the DAS total score, dyadic consensus, dyadic satisfaction, dyadic cohesion, and affective expression subscales were 0.89, 0.81, 0.81, 0.69, and 0.63, respectively.

#### Parenting Self-Esteem

The Parenting Sense of Competence Scale (PSOC; Gibaud-Wallston and Wandersman, 1978, cited in Johnston and Mash, 1989) includes 17 items rated on a six-point Likert scale ranging from 1 (strongly agree) to 6 (strongly disagree), and designed to measure two related but distinct dimensions of parenting self-esteem, i.e., satisfaction with parenting and self-efficacy in the parenting role. Parenting satisfaction is an affective dimension reflecting parenting motivation, frustration, and anxiety (Johnston and Mash, 1989); parenting self-efficacy is as an instrumental dimension involving competence, problem-solving ability, and capability in the parenting role (Ohan et al., 2000). The PSOC has shown an adequate reliability, factor structure, and validity (Ohan et al., 2000). In the present study, in accordance with the Johnston and Mash (1989) method, parents were asked to complete the PSOC while thinking only about one target child in the family (their firstborn). The internal consistency of this measure for our sample was 0.75 for parenting satisfaction and 0.70 for parenting self-efficacy.

### Data Analysis

First, we examined descriptive statistics, gender-related differences in parenting self-efficacy and parenting satisfaction, and bivariate relationships between the measures. We had only two participants with one missing item in the DAS scale (in the DC subscale). In that case, we computed the mean for the non-missing responses. We had no missing data in the ECR-R and PSOC scales. Then we considered the pattern of relationships in our theoretical model using path analysis with the SPSS Amos software (Arbuckle, 2011). Path analysis enables direct and indirect dependence to be tested in a set of variables, providing estimates of the magnitude and significance of the causal connections hypothesized between variables. Path coefficients were estimated using the maximum likelihood method. Each parameter estimate was considered statistically significant if the *t*-test result was *p* < 0.05. At the beginning, we included in the model all direct paths from attachment anxiety and attachment avoidance to parenting self-efficacy and parenting satisfaction, as well as all paths from attachment to dyadic adjustment and from dyadic adjustment to parenting self-esteem. Then, as in the method used by Millings et al. (2012), the model was refined in a series of steps, in which a portion of the model was constrained and the reduction of the model fit verified. If a constraint did not decrease the model fit, then we accepted the simplified model and performed the next step. The following four indices were used to assess the goodness of fit of each model: (a) a chi-square statistic with *p* > 0.05 (i.e., statistically non-significant); (b) a goodness-of-fit index (GFI) above 0.95; (b) a comparative fit index (CFI) above 0.95; a root-mean-square error of approximation (RMSEA) smaller than 0.06. We then performed a bootstrap analysis (based on 2,000 replications) to calculate CIs for path coefficients for the model. Finally, we used a multiple group analysis with no *a priori* hypotheses to check whether the final model was consistent between genders.

## Results

### Effects of Parent’s Gender and Demographic Data on Parenting Self-esteem

First, we used paired *t*-test comparisons on the PSOC scores of mothers and fathers belonging to the same family. Mothers rated their own parenting satisfaction significantly lower than fathers (*t*[117] = 3.37, *p* = 0.001), whereas there were no differences between the two on the parenting self-efficacy scale (*t*[117] = 0.79, *n.s.*).

To check the effects on PSOC scores of the child’s gender and its interaction with the parent’s gender, we conducted two analyses of variance, with parenting satisfaction and self-efficacy as dependent variables, and the child’s and parent’s gender as between-subjects factors. As expected, we found a significant effect of the parent’s gender on parenting satisfaction (*F*[1,232] = 8.11, *p* = 0.005), while the child’s gender (*F*[1,232] = 0.001, *n.s.*) and the interactions between factors were not significant. No significant effects or interactions were found for parenting self-efficacy.

Then, we separately analyzed the mothers’ and fathers’ ratings, calculating Pearson’s bivariate correlations between the PSOC measures and the demographic data (the parent’s age and years of formal education, duration of the couple’s relationship, the child’s age, and the number of children). Parenting satisfaction showed a significant negative correlation with the child’s age, for both mothers (*r* = -0.21, *p* = 0.028) and fathers (*r* = -0.19, *p* = 0.037). Satisfaction also correlated negatively with the number of children in the family, but only for fathers (*r* = -0.18, *p* = 0.047). Satisfaction was not related with either parent’s age or formal education, or the duration of their relationship.

Fathers’ parenting self-efficacy correlated negatively with the parent’s age (*r* = -0.26, *p* = 0.005), the child’s age (*r* = -0.24, *p* = 0.008), and the number of children (*r* = -0.23, *p* = 0.010), but was unrelated with formal education or duration of the couple’s relationship. Mothers’ parenting self-efficacy was independent of all these variables.

Correlations computed between mothers’ and fathers’ PSOC scores showed significant relations for parenting satisfaction (*r* = 0.23, *p* = 0.011), and parenting self-efficacy (*r* = 0.20, *p* = 0.027). The satisfaction and self-efficacy scores correlated significantly for both mothers (*r* = 0.39, *p* < 0.001), and fathers (*r* = 0.54, *p* < 0.001).

Before conducting the path analysis, we computed Pearson’s bivariate correlations for adult attachment, dyadic adjustment, and parenting self-esteem (**Table 1**). As expected, this correlation analysis showed that the two dimensions of parenting self-esteem (satisfaction and self-efficacy) correlated negatively with the attachment measures in both parents, and positively with dyadic adjustment. Attachment anxiety was the predictor variable showing the strongest correlation with parenting satisfaction, and total score on the DAS with parenting self-efficacy. Lastly, we compared mothers’ and fathers’ scores of attachment dimensions and dyadic adjustment using independent samples *t*-test. Mothers scored significantly higher on attachment anxiety (*M* = 2.69, *SD* = 0.93) than fathers (*M* = 2.45, *SD* = 0.73); *t*(234) = -2.19, *p* = 0.029. There were no significant differences in the attachment avoidance (*t*[234] = 1.42, *n.s.*) and dyadic adjustment scores (*t*[234] = -0.13, *n.s.*) between mothers and fathers.

**Table 1:** Pearson’s correlation, mean scores, and standard deviation for measures of adult attachment, dyadic adjustment, and parenting self-esteem.

Measure	1	2	3	4	5	*M* (fathers)	*SD* (fathers)
(1) Attachment anxiety (ECR-R)	–	0.48^∗∗^	-0.50^∗∗^	-0.63^∗∗^	-0.26^∗∗^	2.45	0.74
(2) Attachment avoidance (ECR-R)	0.65^∗∗^	–	-0.54^∗∗^	-0.40^∗∗^	-0.26^∗∗^	2.02	0.71
(3) Dyadic adjustment (DAS total score)	-0.62^∗∗^	-0.66^∗∗^	–	0.40^∗∗^	0.26^∗∗^	119.41	12.64
(4) Parenting satisfaction (PSOC)	-0.43^∗∗^	-0.27^∗∗^	0.29^∗∗^	–	0.39^∗∗^	44.48	5.17
(5) Parenting self-efficacy (PSOC)	-0.27^∗∗^	-0.23^∗^	0.32^∗∗^	0.54^∗∗^	–	30.71	4.34
*M* (mothers)	2.69	1.88	119.64	42.22	31.50		
*SD* (mothers)	0.93	0.72	14.27	6.48	4.81		

*Results for mothers (n = 118) are shown below the diagonal; results for fathers (n = 118) above the diagonal. ^∗^p < 0.05; ^∗∗^p < 0.01.*

### Path analysis

Initially, the overall model of parenting self-esteem shown in **Figure 1** (Model 1) was tested. Child’s age was included in the model as covariate, because in the preliminary analyses it resulted significantly associated with parenting self-esteem in both mothers and fathers (parent’s age and number of children were not included because they were both significantly correlated with child’s age). Model 1 showed only a partial fit with the data: χ^2^(4) = 9.97, *p* = 0.041; GFI = 0.986; CFI = 0.984; RMSEA = 0.080 (90% CI [0.015, 0.143]). The path from child’s age to parenting satisfaction was significant (β = -0.17, *p* < 0.001) and thus it was included in all the subsequent steps of the model refinement. Inspecting the direct paths from attachment to parenting self-esteem, the only significant direct effect was the negative path from attachment anxiety to parenting satisfaction (β = -0.48, *p* < 0.001).

Next, we verified the direct associations between attachment and parenting self-esteem by constraining all attachment paths to parenting self-esteem to zero (Model 2). This model fitted significantly worse than Model 1, Δχ^2^(4) = 56.48, *p* < 0.001, and did not meet good-fit criteria: χ^2^(8) = 66.45, *p* < 0.001, GFI = 0.922; CFI = 0.840; RMSEA = 0.176 (90% CI [0.139, 0.217]).

Therefore, we next fixed to zero all attachment paths to parenting self-esteem except that from attachment anxiety to parenting satisfaction (Model 3). This step did not decrease model fit compared with Model 1, Δχ^2^(3) = 3.85, *p* > 0.05, and Model 3 met satisfactory fit criteria: χ^2^(7) = 13.83, *p* = 0.054; GFI = 0.982; CFI = 0.981; RMSEA = 0.064 (90% CI [0.000, 0.114]). Examining the coefficients from dyadic adjustment and parenting self-esteem, it resulted that dyadic adjustment significantly predicted parenting self-efficacy (β = 0.30, *p* < 0.001) but not parenting satisfaction (β = -0.03, *p* > 0.05).

Consequently, in Model 4 we examined whether there were direct paths from dyadic adjustment to parenting self-efficacy and parenting satisfaction, by constraining both paths to zero. This model fitted significantly worse, Δχ^2^(2) = 21.79, *p* < 0.001, indicating that a meaningful effect of dyadic adjustment was present in the data.

**FIGURE 2:**
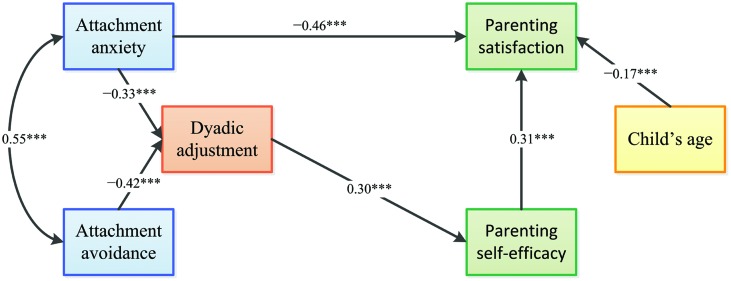
**Final path model of the effects of adult attachment and dyadic adjustment on parenting self-efficacy and parenting satisfaction.** Path coefficients are standardized structural coefficients; ^∗∗∗^*p* < 0.001.

Finally, we included in the model the direct path from dyadic adjustment to parenting self-efficacy, fixing to zero the path from dyadic adjustment and parenting satisfaction (Model 5). This model did not fit significantly worse than Model 3, Δχ^2^(1) = 0.26, *p* > 0.05, and model fit was good: χ^2^(8) = 14.09, *p* = 0.079; GFI = 0.982; CFI = 0.983; RMSEA = 0.057 (90% CI [0.000, 0.105]). Therefore, we considered Model 5 as our final model for interpretation (see **Figure 2**).

In the final model, higher levels of attachment anxiety were associated with lower levels of parenting satisfaction (β = -0.46, *p* < 0.001). Higher levels of attachment anxiety (β = -0.33, *p* < 0.001) and attachment avoidance (β = -0.42, *p* < 0.001) had a negative impact on dyadic adjustment. Dyadic adjustment positively influenced parenting self-efficacy (β = 0.30, *p* < 0.001) which, in turn, increased parenting satisfaction (β = 0.31, *p* < 0.001). Child’s age was negatively correlated with parenting satisfaction (β = -0.17, *p* < 0.001).

The squared multiple correlations indicated that our model could account for 44% of the variance in dyadic adjustment, 9% in parenting self-efficacy, and 39% in parenting satisfaction.

The significance of the (indirect) mediating effects of dyadic adjustment and parenting self-efficacy in the final model were tested using the bootstrap estimation procedure in AMOS (specifying a bootstrap sample of 2000). **Table 2** shows the indirect effects and their associated 95% confidence intervals. None of the estimated values of the indirect paths overlapped with zero, indicating that both attachment anxiety and attachment avoidance have a significant indirect effect on both measures of self-esteem, mediated by the effect of dyadic adjustment.

**Table 2:** Final model: standardized indirect effects and 95% confidence intervals based on 2000 bootstrap replications.

Model pathways	Estimated	95% CI
Attachment anxiety → Dyadic adjustment → Parenting self-efficacy	-0.10^a^	[-0.16, -0.05]
Attachment anxiety → Dyadic adjustment → Parenting self-efficacy → Parenting satisfaction	-0.03^a^	[-0.06, -0.01]
Attachment avoidance → Dyadic adjustment → Parenting self-efficacy	-0.12^a^	[-0.18, -0.08]
Attachment avoidance → Dyadic adjustment → Parenting self-efficacy → Parenting satisfaction	-0.04^a^	[-0.07, -0.02]
Dyadic adjustment → Parenting self-efficacy → Parenting satisfaction	0.09^a^	[0.05, 0.15]

*^a^Empirical 95% confidence intervals do not overlap with zero.*

Finally, we ran a multi-group analysis to see whether the path coefficients differed significantly between mothers and fathers. We compared the first model (which allowed for the structural paths to vary across genders) with the second (which constrained the regression paths to remain the same for mothers and fathers) in order to identify any gender-related differences. The non-significant chi-square differences between the two models, Δχ^2^(6) = 12.18, *p* > 0.05, suggest that the final model did not differ by gender. In other words, multi-group analysis indicated that gender did not moderate the association between the variables in the combined final model.

## Discussion

The purpose of this study was to examine the links between adult attachment insecurities, dyadic adjustment, and parenting self-esteem (i.e., parenting self-efficacy and parenting satisfaction) in a sample of non-clinical parents, and to develop and validate an integrative theoretical model of these connections.

Parenting self-efficacy and parenting satisfaction are crucial psychological components of parenting self-esteem that affect both the parent’s personal well-being and the parent’s relationship with his/her child. Adults who feel effective and satisfied as parents are more likely to be behave appropriately with their children, and to provide an adequate quality of care (Dix and Grusec, 1985; Johnston, 1996; Bugental and Johnston, 2000; Sigel and McGillicuddy-Delisi, 2002; Rubin and Chung, 2006). The importance of parents’ beliefs concerning their parenting role has been seen in several phases of a family’s life cycle, from the prenatal phase of the transition to parenthood (Palkovitz, 1992) to the offspring’s adulthood (Schofield et al., 2014).

There is now convincing empirical evidence of parents’ adult attachment influencing many facets of parenting and directly and indirectly affecting parents’ behavior, emotions, and cognitions (Jones et al., 2015). So far, however, the relatively small number of studies on the influence of adult attachment styles – assessed by means of self-report measures focusing on aspects of parents’ self-esteem such as parenting self-efficacy and parenting satisfaction – have yielded a complex and unclear picture. There is some evidence of adult attachment styles affecting parents’ self-esteem, and their self-efficacy in particular, while findings concerning parenting satisfaction are less consistent and more difficult to interpret. Such preliminary findings are also difficult to extend to the general (non-clinical) population of families because they have often concerned parents at risk or facing challenging conditions or difficulties. It is also noteworthy that the extant literature fails to consider the plausible mediating role of dyadic adjustment in families where both parents are present.

Based on these premises, we analyzed the determinants of parenting self-efficacy and parenting satisfaction in a sample of non-clinical families belonging to the general normative population, with a firstborn child aged from 0 to 6 years. We developed an integrative model linking attachment insecurities to parenting self-efficacy and parenting satisfaction, taking the mediating influence of the couple’s dyadic adjustment into account. To obtain a complete picture of the couples involved, we only considered families in which both parents – married or living together – participated in the study.

In the current study, fathers reported higher levels of parenting satisfaction than mothers, whereas there was no difference between the parents’ self-efficacy scores. These results replicate the findings reported by several researchers who found that fathers were more satisfied with their parenting role than mothers (Johnston and Mash, 1989; Rogers and Matthews, 2004; Gilmore and Cuskelly, 2009). According to Johnston and Mash (1989), an explanation for this may lie in the greater emphasis that fathers put on playing activities in their parenting role (Lamb, 1976), in contrast with the more instrumental and demanding nature of the mother’s parenting role.

Additionally, parenting satisfaction was negatively influenced by child’s age, for both mothers and fathers, i.e., both parents were more satisfied as parents when their children were younger, and they became less satisfied as their child grew older. This effect had already been found in some previous studies (Mash and Johnston, 1983; Rogers and Matthews, 2004), but not in others (Johnston and Mash, 1989; Ohan et al., 2000; Gilmore and Cuskelly, 2009), so this issue needs to be further investigated.

We also found that fathers’ parenting satisfaction correlated negatively with the number of children in the family, whereas – consistently with previous research (Johnston and Mash, 1989; Ohan et al., 2000; Rogers and Matthews, 2004; Gilmore and Cuskelly, 2009) – parenting satisfaction was found unaffected by any of the other variables considered, such as age, years of formal education, and duration of the couple’s relationship, for mothers or fathers.

Lastly, when considering the influence of such potential covariate or moderator variables on parenting self-efficacy, our findings showed that fathers who were younger, or had fewer children, or their children were younger, tended to perceive themselves as more effective in managing parenting tasks than older fathers, with older children, or in families with a larger number of children. These outcomes are consistent with previous reports of the father’s role being less well-articulated and defined by social convention than the mother’s (Belsky et al., 1991), and less stable in the father’s involvement over time (Coley and Chase-Lansdale, 1999). It has been reported that, as their children grow up, fathers significantly reduce their level of involvement in absolute terms (Yeung et al., 2001; Parke, 2002), and this lesser involvement may result in a diminished sense of parenting self-efficacy.

In the preliminary correlation analyses, we found that attachment anxiety and attachment avoidance correlated negatively with dyadic adjustment and parenting self-esteem in both mothers and fathers. On the other hand, dyadic adjustment correlated positively with parenting self-efficacy and parenting satisfaction in both genders. In other words, consistently with theoretical expectations, attachment insecurities were significant *negative* predictors of couple quality and parenting self-esteem, and dyadic adjustment was a significant *positive* predictor of parenting self-esteem.

Path analysis revealed a theoretical integrative model in which dyadic adjustment mediates the influence of attachment insecurities on parenting self-efficacy and, at the same time, attachment orientations directly affect parenting satisfaction, which in turn is negatively influenced by child’s age. The multiple group comparison also showed that the pattern of relationships between the variables was the same for mothers and fathers, which goes to show that adult attachment and dyadic adjustment may be equally important for women and men in terms of their parenting self-esteem.

Based on our results, we can draw some conclusions consistent with previous studies on the influence of attachment orientations on couple quality and parenting.

First, as hypothesized in our model, higher levels of attachment insecurity (attachment anxiety and attachment avoidance) are associated with lower levels of dyadic adjustment in the couple’s relationship. This association is consistent with reports of attachment insecurities negatively affecting dyadic adjustment and dyadic satisfaction: insecure attached people report lower levels of couple adjustment and satisfaction in almost every phase of the family life cycle, and in various parenting conditions (Collins and Read, 1990; Carnelley et al., 1994, 1996; Collins, 1996; Frazier et al., 1996; Jones and Cunningham, 1996; Whisman and Allan, 1996; Cozzarelli et al., 2000; Frei and Shaver, 2002; Schmitt, 2002; Steiner-Pappalardo and Gurung, 2002; Shi, 2003; Kachadourian et al., 2004; Sumer and Cozzarelli, 2004; Williams and Riskind, 2004; Shaver et al., 2005; Calvo et al., 2015).

The above finding may indicate that attachment security (i.e., low anxiety and low avoidance) is associated with a greater dyadic adjustment. In fact, the literature indicates that individuals more secure in their attachment generally show higher levels of satisfaction with their relationship and are better able to handle relationship stress without experiencing a loss of relationship quality (Amir et al., 1999; Rholes et al., 2001). More in general, this path confirms the important influence of adult attachment on many aspects of a couple’s relationship (Mikulincer and Shaver, 2007). It has been well-established that secure individuals can facilitate the consolidation of a lasting positive relationship with their partners (Morgan and Shaver, 1999), whereas attachment insecurities are associated with less constructive attitudes and beliefs, and a dyadic behavior that may interfere with the construction of a couple’s relationship as a secure base. As Mikulincer and Shaver (2007) showed in their review, people with insecure attachment styles report less intimacy (Knobloch et al., 2001; Treboux et al., 2004; Whiffen, 2005), lower levels of commitment (Tucker and Anders, 1999; Steiner-Pappalardo and Gurung, 2002; Treboux et al., 2004), and more difficulties with communication (Fitzpatrick et al., 1993; Feeney et al., 1994; Feeney, 1995, 1999), and with managing interpersonal conflict in their relationships (Feeney, 1994; Feeney et al., 1994; Roberts and Noller, 1998; Shi, 2003; Marchand, 2004; Marchand et al., 2004) than secure individuals.

Second, our model confirmed the hypothesis that parenting self-efficacy is affected directly by dyadic adjustment on the one hand, and indirectly by attachment dimensions on the other. As expected, parents’ attachment insecurities impaired the quality of the couple’s relationship, which in turn was associated with lower levels of parenting self-efficacy.

We postulated a causal connection between dyadic adjustment and parenting self-efficacy in accordance with the spillover hypothesis (Erel and Burman, 1995), because parenting self-efficacy is considered an instrumental dimension of parenting. The spillover hypothesis suggests that parents who have satisfying and supportive relationships as a couple will be more sensitive to the needs of their child (Easterbrooks and Emde, 1988), and experience less discord concerning discipline and fewer inconsistencies in their parenting (Erel and Burman, 1995). These aspects augment their personal instrumental feeling of being competent, capable of solving problems, and familiar with their parenting role (Johnston and Mash, 1989). On the other hand, according to the same spillover hypothesis, a couple’s negative relationship may lead the parents to engage in stressful and dysfunctional interactions that leave them irritable and emotionally drained. They consequently become less efficacious as parents, less attentive and sensitive to their child’s needs (Easterbrooks and Emde, 1988; Erel and Burman, 1995). Numerous studies have shown that a good relationship between the two parents – in terms of agreement, intimacy, warm interaction, and effective communication – can enhance parenting practices and have a significant positive impact on parenting self-efficacy (Belsky, 1984; Goldberg and Easterbrooks, 1984; Cox et al., 1989; Howes and Markman, 1989; Simons et al., 1992, 1993; Kerig et al., 1993; Erel and Burman, 1995; Cowan et al., 1996; Holloway et al., 2005; Schoppe-Sullivan et al., 2007; Suzuki, 2010), whereas parents’ conflictual relationships with one another and hostile communication are negatively associated with optimal parenting behavior (Kerig et al., 1993).

As expected, parenting self-efficacy was positively associated with parenting satisfaction. Parents who have more positive perceptions of their efficacy as parents tend to experience higher levels of satisfaction with their parenting role. It has been shown that efficacious parents are likely to be more at ease and effective in dealing with parenting problems, and this reflects positively on the satisfaction they derive from being parents, whereas anxiety, stress and depression coincide with lower levels of self-efficacy and consequently less parenting satisfaction (Coleman and Karraker, 1997).

Taking these findings together, our model identified a significant indirect negative effect of attachment insecurities on parenting satisfaction, considering the mediating effect of both dyadic adjustment and parenting self-efficacy. Security of attachment is associated with higher levels of dyadic adjustment, which reinforces parenting self-efficacy, increasing parenting satisfaction as a result. It is noteworthy that this indirect path linking attachment, dyadic adjustment, parenting self-efficacy and parenting satisfaction applied to both parents. This finding is particularly relevant if we consider the repercussions that satisfaction with fatherhood and caregiving have on the father’s involvement. Several studies have reported significant links between fathers’ satisfaction with their relationship with their partners and their participation in childcare (Levy-Shiff and Israelashvili, 1988; Volling and Belsky, 1991; Coley and Chase-Lansdale, 1999), and there is evidence of the couple’s support for each other being more crucial to fathers’ than to mothers’ adequate parenting (Parke, 2002). Our model suggests that adult attachment is indirectly involved in this pathway of influences and may therefore play a relevant part in fathers’ involvement too. Further research is warranted to confirm this implication of our findings.

Lastly, the results emerging from our model suggest that attachment anxiety has a direct negative association with parenting satisfaction. These results are consistent with previous reports of attachment anxiety being more associated with poor affect regulation and emotional control, and distress, than in the case of secure and avoidant attachment (Cooper et al., 1998; Feeney, 1999).

According to adult attachment theory, internal working models are thought to influence not only how individuals organize their behavior but also how they perceive, attend to, and process information of emotional significance (Niedenthal et al., 2002; Feeney and Cassidy, 2003; Fraley et al., 2006). Individuals with anxious attachment have been described as characterized by a chronic activation of the attachment system and by a major use of hyperactivating strategies in attachment-related situations (Cassidy and Kobak, 1988; Main, 1990; Shaver and Mikulincer, 2002; Mikulincer et al., 2003; Collins et al., 2006; Mikulincer and Shaver, 2007). These strategies may have a negative impact on emotional information processing, amplifying the individual’s distress, and ultimately affecting their self-image and satisfaction with themselves (Mikulincer and Shaver, 2007). Consistently with theoretical expectations, attachment anxiety levels have been found to correlate inversely with satisfaction, not only with relationships in a couple, but also with life generally (Kirchmann et al., 2013), and even with the outcome of nasal plastic surgery (Saragusty et al., 2011). This link may also reflect anxious individuals’ persistent feeling that they are not getting enough out of their relationships and want more, even in relationships with their children. They may feel they are not as close as they would like to be to their children and this may reduce satisfaction.

Contrary to our hypothesis, we found no significant direct negative path from attachment avoidance to parenting self-esteem. However, our model revealed a significant indirect effect linking attachment avoidance to parenting satisfaction, mediated by dyadic adjustment and parenting self-efficacy. From a theoretical standpoint, avoidant attachment is characterized by different emotional regulation strategies from anxious attachment. In the former, such strategies are termed deactivating (Cassidy and Kobak, 1988; Shaver and Mikulincer, 2002), and include creating an emotional distance from others in response to discomfort with interpersonal dependence (Mikulincer et al., 2003), the use of compulsive self-reliance, the suppression of distressing cognitions and memories (Shaver and Mikulincer, 2002; Gross and John, 2003; Mikulincer and Shaver, 2007; Velotti et al., 2015), and the minimization of negative feedback from the outside environment in order to maintain a positive self-image (Bartholomew, 1990; Bartholomew and Horowitz, 1991). As a result, the parenting self-esteem of avoidant individuals is less likely to suffer from negative self-perceptions deriving from a maladaptive emotional regulation.

To conclude, our findings are consistent with attachment theory and the related literature. They confirm the importance of using implicit dimensions of attachment, as well as global attachment classifications, to better understand an adult’s psychological functioning (Salcuni, 2015), and better define their parenting profile (De Palo et al., 2014), particularly when considering such complex psychological traits as parenting self-esteem.

The present study has some limitations that need to be considered. First, our data was of a cross-sectional nature, and this hampered any effort to interpret the direct and mediating effects in a causal sense. Longitudinal investigations are needed to assess the processes underlying the associations in our model. Second, we only considered self-report questionnaires, which are vulnerable to same-source bias. Future directions of research should address this limit using different measures of parental self-esteem and integrating self-report assessments with qualitative analysis of in-depth interviews about parental cognitions and beliefs. Third, in this study we did not include any measure of parents’ depression. In future work, it will be important to consider also this dimension, given the links between depression and parenting self-efficacy and satisfaction (Caldwell et al., 2011; Kohlhoff and Barnett, 2013). Finally, the current study included only parents of 0–6 years-old children. Further research should also examine parents of older children to investigate how parental self-esteem is influenced by attachment orientations and dyadic adjustment, in other child developmental stages, such as middle and late childhood or adolescence.

Despite these limitations, to the best of our knowledge, this is the first study to examine the connections among adult attachment insecurities, dyadic adjustment, and parenting self-esteem in a normative sample of parents, and to develop an integrative model of these links taking the mediating role of dyadic adjustment with the partner into account. Our sample was relatively large and our findings may be relevant to preventive, empowering, and clinical interventions. In fact, we identified dyadic adjustment as a potential “malleable mediator” (Fraser and Galinsky, 2010), a factor on which action may be taken in an effort to mitigate the negative influence of attachment insecurities on parenting self-esteem. This finding could have crucial practical implications because professionals could enhance parenting self-efficacy more effectively by intervening on couple quality. According to Jones and Prinz (2005), parenting self-efficacy should be considered one of the targets for prevention programs to improve the well-being of parents and children.

## Author Contributions

Design and conceptualization of the study: VC. Statistical analysis and interpretation of the data: VC. Drafting and revising the manuscript: VC, FB. Final approval of the version to be published: VC, FB. Agreement to be accountable for all aspects of the work in ensuring that questions related to the accuracy or integrity of any part of the work are appropriately investigated and resolved: VC, FB.

## Conflict of Interest Statement

The authors declare that the research was conducted in the absence of any commercial or financial relationships that could be construed as a potential conflict of interest.
